# The Anatomy of Iconicity: Cumulative Structural Analogies Underlie Objective and Subjective Measures of Iconicity

**DOI:** 10.1162/opmi_a_00162

**Published:** 2024-09-23

**Authors:** Stella Punselie, Bonnie McLean, Mark Dingemanse

**Affiliations:** Centre for Language Studies, Radboud University; Department of Linguistics and Philology, Uppsala University

**Keywords:** iconicity, structure-mapping, psycholinguistics, sound symbolism, ideophones

## Abstract

The vocabularies of natural languages harbour many instances of iconicity, where words show a perceived resemblance between aspects of form and meaning. An open challenge in this domain is how to reconcile different operationalizations of iconicity and link them to an empirically grounded theory. Here we combine three ways of looking at iconicity using a set of 239 iconic words from 5 spoken languages (Japanese, Korean, Semai, Siwu and Ewe). Data on guessing accuracy serves as a baseline measure of probable iconicity and provides variation that we seek to explain and predict using structure-mapping theory and iconicity ratings. We systematically trace a range of cross-linguistically attested form-meaning correspondences in the dataset, yielding a word-level measure of cumulative iconicity that we find to be highly predictive of guessing accuracy. In a rating study, we collect iconicity judgments for all words from 78 participants. The ratings are well-predicted by our measure of cumulative iconicity and also correlate strongly with guessing accuracy, showing that rating tasks offer a scalable method to measure iconicity. Triangulating the measures reveals how structure-mapping can help open the black box of experimental measures of iconicity. While none of the methods is perfect, taken together they provide a well-rounded way to approach the meaning and measurement of iconicity in natural language vocabulary.

## INTRODUCTION

Iconicity, a perceived resemblance between aspects of form and meaning, has gained attention in recent years as a key aspect of natural languages (Perniss et al., 2010; Vigliocco & Kita, 2006). With a growing amount of work assessing its role in language emergence (Cuskley & Sommer, 2023), language change (Erben Johansson, 2024), word learning (Yoshida, 2012) and communication (Laing, 2019), it is more important than ever to provide proper theoretical and empirical grounding for the concept of iconicity.

A formidable challenge that has plagued iconicity studies at least since Plato’s *Cratylus* is that no language user can avoid having an opinion about how form and meaning may or may not fit each other. This means that the topic of iconicity has always attracted equal measures of far-fetched speculation (Magnus, 1999; Peck, 1886) and flippant scepticism (Newmeyer, 1992; Whitney, 1874). The resulting rhetorical skirmishes, though entertaining, have tended to obscure scientific progress made along more moderate avenues. Jespersen captured this paradox well when he objected that “much of what people ‘hear’ in a word appears to me fanciful and apt to discredit reasonable attempts at gaining an insight into the essence of sound symbolism” (Jespersen, 1922).

There are various ways to avoid fanciful speculation while still taking iconicity seriously. A method that goes back to Jespersen’s era is the experimental approach to sound symbolism, which asks people to make guesses about form-meaning associations, either of made-up words (Fischer-Jørgensen, 1978; Sapir, 1929; Styles & Gawne, 2017) or of actual words from natural languages (Brown et al., 1955; Nygaard et al., 2009; Slobin, 1968). Such work provides evidence of persistent biases in how form and meaning are associated, without worrying about how exactly these associations may work. A more recent approach is to ask people for subjective judgements about the degree of form-meaning fit (Vinson et al., 2008; Winter et al., 2023). Such rating tasks are on the rise as they provide a more scalable approach to collecting data on iconicity for large numbers of words, albeit without directly addressing what intuitions are being tapped into exactly. In the visual modality, the latest work in this vein has shown that the subjective iconicity of signs is modulated by language-specific and bodily experience (Börstell, 2024; Occhino et al., 2017; Ortega et al., 2019; Sevcikova Sehyr & Emmorey, 2019). In spoken language vocabulary, subjective iconicity ratings correlate with measures of word frequency, structural markedness and age of acquisition (Winter et al., 2023). All of these methods, however, leave open the fundamental question of how, ultimately, people are able to produce reasonable guesses or consistent subjective ratings of word-level iconicity.

Here we aim to address that question with a three-pronged approach that combines a first-principles analysis of iconicity as structure-mapping (Emmorey, 2014; Gentner, 1983; Taub, 2001) with multiple sources of data. We use experimental data on word guessability as a measure of the degree to which form can suggest aspects of meaning; collect iconicity ratings as a measure of subjective form-meaning fit; and ground these two measures in a principled appraisal of cross-linguistically attested structural analogies between form and meaning. The strength of this approach lies in triangulation, with the three methods enriching and constraining each other.

## CONCEPTUAL FOUNDATIONS OF ICONICITY

If there is such a thing as iconicity in natural language, we ought to be able to not just measure it—as we do in guessing and rating tasks—but also explain it in a way that is predictive of task outcomes and real-world measures. On the whole, experimental work on iconicity has long shown effects consistent with iconicity, but has been somewhat reticent in grounding these effects directly and systematically in a theory of iconicity that can explain them in principled ways (Motamedi et al., 2019; Sidhu & Pexman, 2018).

To be sure, theoretical accounts of iconicity have long existed, mostly in parallel lines of work in linguistics and semiotics (Ahlner & Zlatev, 2010; Peirce, 1955; Waugh, 1994). Wilhelm Wundt proposed that *Lautbilder* or ‘sound images’ worked by creating acoustic and articulatory depictions of sensory scenes (Wundt, 1922). Building on this, Westermann examined lexical material from a dozen West-African languages and proposed a number of cross-linguistically attested form-meaning associations (Westermann, 1927, 1937). Scholars working on Quechua and Japanese have proposed a range of phonosemantic mappings found in lexical classes of vivid sensory words known as ideophones or mimetics (Hamano, 1998; Nuckolls, 1996). Work on sign language iconicity has described the many ways in which visible articulators can suggest meaning (Mandel, 1976; Sallandre & Cuxac, 2001; Wilcox, 2004). Work in semiotics has formulated principles of iconic mappings across sensory modalities (Ahlner & Zlatev, 2010; Peirce, 1955). From this work across languages and modalities we know that iconicity is driven by perceptual analogies between aspects of form and meaning. However, as much of this work builds off of well-chosen examples rather than systematic sweeps of iconic vocabulary, it has long been vulnerable to a converse criticism, namely that its theoretical forays lack systematic empirical grounding.

Here we aim to address both challenges—of grounding experimental effects in a theory of iconicity, and of grounding a theory of iconicity in empirically attested form-meaning associations—by means of triangulation. For this, we need a theoretical approach that can extend in both directions: one that can explain qualitative observations and make quantitative predictions. A promising approach in this regard is one that sees iconicity as a form of structure-mapping (Emmorey, 2014; Gattis, 2004; Meir, 2010; Taub, 2000; Tufvesson, 2011).

### Structure-Mapping and Natural Language Iconicity

**Figure 1.**
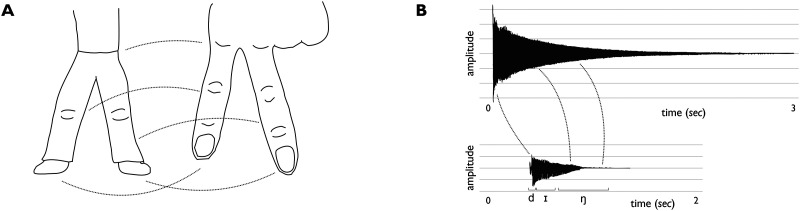
Two examples of iconic signs, with lines indicating structural correspondences between source and target. A: a pair of human legs (and their movement) can be iconically represented by two fingers. B: Onset, amplitude envelope and resonance of the sound of a bell can be iconically represented by structurally similar acoustic and temporal properties of the word ding [dɪŋː] in English. (Adapted from Taub, 2001: 22, 24 © Cambridge University Press. Reproduced with permission of The Licensor through PLSclear.)

Structure-mapping theory deals in structure-preserving correspondences between two domains (Gentner, 1983). In the case of iconicity, these correspondences are structural analogies between aspects of form and meaning. Figure 1 shows examples of iconicity in the visual and auditory domain (from Taub, 2001, pp. 22–25). When we say that two legs can be iconically represented by two fingers, we mean that aspects of the perceived structure of the legs can be seen to correspond to aspects of the perceived structure of the fingers. Likewise, when we say that the reverberating sound of a bell can be iconically represented by a word like *ding* [dɪŋː] we mean that aspects of the perceived structure of the one acoustic event can be seen to correspond to aspects of the perceived structure of the other.

The process of setting up structural analogies (in short, structure-mapping) has some features of particular relevance for natural language iconicity. First, structure-mapping is necessarily *selective*: the focus is not on constructing a perfect replica, but on setting up a set of correspondences that preserve salient structure across source and target, or form and meaning, or sign and signified. This provides room for a degree of semiotic and linguistic diversity (Occhino et al., 2020). In the ASL sign for ‘walking’, the fingers don’t stand for the legs, as in Figure 1; instead, the hands depict the feet and their movement. The fact that onomatopoeia for barking and other animal sounds differ across languages (within limits) is also a result of this, and shows there are considerable degrees of freedom in structure-mapping.

Second, *structural properties* of source and target shape and constrain possible iconic mappings. To depict the legs, the fingers and hands are arguably better choices than body parts that do not come in multiples; and a bell is better depicted by sonorant sounds than by voiceless fricatives. To understand natural language iconicity, then, we must attend closely to aspects of both form and meaning. This can help explain which semantic domains are more or less likely to afford iconicity (Nuckolls, 2019; Van Hoey, 2022), and how this may differ across modalities (Keränen, 2023; Thompson et al., 2020). It also means that amidst the expected cross-linguistic diversity, we should be able to find cross-linguistic commonalities in iconic mappings.

Third, structural correspondences can be *cumulative*. The index and middle fingers not only come in twos like the legs, but they are also adjacent, have joints that can be seen to correspond to the knees, and can move somewhat like the legs (Figure 1A). It is probably the accumulation of these structural correspondences that makes us feel that these two fingers are more effective resources for depicting a pair of legs than, say, two nonadjacent fingers, or paired body parts like ears or eyes that do not afford the same amount of structural correspondences. Likewise for the bell (Figure 1B): a word form that expresses the onset (e.g., with a plosive sound), the resonance (e.g., with a sonorant) as well as the singular aspectual structure (e.g., with a single syllable) would likely be judged more iconic than word forms that tap into only one or two such possible correspondences.

A final consequence of the partial and schematic nature of structure-mapping is that not all aspects of source and target domain need to be—or indeed could be—in correspondence. Plenty of *non-alignable differences* are expected: aspects of one situation that have no correspondence in the other (Gentner & Markman, 1997). For instance, the joints of legs and fingers do not match up perfectly and clothing is not represented at all; these aspects are not part of the set of structural correspondences. And not every aspect of the phonetic make-up of English *ding* [dɪŋː] is in structural correspondence with the sonic event evoked. The nasal coda is useful for evoking a resonant sound, but its velar place of articulation may be just as much a matter of articulatory ease or social convention. We can expect all ‘iconic’ signs in natural languages to have such non-alignable or arbitrary aspects.^1^ It follows that signs can differ in the degree of iconicity they exhibit, but few if any signs can be said to be exclusively iconic (Capirci et al., 2022; Hodge & Ferrara, 2022).

So far, our cherry-picked examples could still be vulnerable to Jespersen’s charge of fanciful speculation. Two tools can be used to strengthen structure-mapping accounts of natural language iconicity: cross-linguistic attestation and empirical verification. These tools make structure-mapping accountable to diverse data and open it up to falsification, two important properties that just-so stories and fanciful speculations lack. By *cross-linguistic attestation* we mean that structure-mapping accounts of iconicity gain credibility if they are not based on a small number of well-chosen words but instead capture patterns found across diverse languages.^2^ By *experimental verification* we mean that structure-mapping accounts must show a transparent relation to experimental measures that tap into iconicity, such as results from rating, guessing and learning tasks.

If words count as highly iconic according to a structure-mapping account, yet turn out not to be guessable above chance and not to be rated as highly iconic, structure-mapping can hardly be said to contribute to our understanding of iconicity. If, on the other hand, structure-mapping can help predict which words are most guessable and rated as most iconic, this would mean that structure-mapping helps us identify some of the cues that facilitate people’s intuitions about word meaning and ratings of subjective iconicity. It would be positive evidence for structure-mapping as an empirically grounded theory of natural language iconicity.

### Our Empirical Focus: Spoken Language Ideophones

The theory of iconicity as structure-mapping has been relatively prominent in the analysis of iconicity in the visual-spatial modality (Capirci et al., 2022; Emmorey, 2014; Keränen, 2023; Taub, 2001), but less so in spoken languages (Taub, 2000; Thompson & Do, 2019; Tufvesson, 2011). This is in part due to a widespread assumption that speech can only depict sound, making it difficult to see how iconicity in speech could ever go beyond limited cases like the onomatopoetic ‘ding’ above. However, speech offers a rich and multidimensional bundle of articulatory and acoustic events, and it provides ample structure for building a broader range of perceptual analogies (Ahlner & Zlatev, 2010; Bühler, 1934; Nuckolls, 1996; von Humboldt, 1836; Waugh, 1994; Westermann, 1927).

One place where these broader forms of spoken language iconicity comes to the fore is in *ideophones*: vivid sensory words found in the form of an open lexical class in many of the world’s languages (Dingemanse, 2019). Ideophones as a cross-linguistic category are noteworthy for the way they employ articulatory and acoustic properties in the service of depicting sensory scenes (Diffloth, 1972; Nuckolls, 1996; Westermann, 1905). Their meanings cover a wide range of sensory imagery, from sound and movement to shape, texture, and other sensory perceptions (McLean, 2020). Experimental studies have found them to be, on average, guessable above chance—an indication of their possible iconicity (Fischer-Jørgensen, 1978; McLean et al., 2023).

Our point of departure is a set of 239 ideophones tested for guessability in two prior studies (Dingemanse et al., 2016; Lockwood et al., 2016). Crucially, they present a gradient of guessability, ranging from ideophones guessed at chance level or worse to ideophones guessed far above chance level. The variability is partly regimented by semantic domain: ideophones for sounds were guessed significantly better than other domains. The variation in guessability is what we use as a lever to gain insight into what drives iconicity and how it is distributed over the lexicon.

### Predictions

The theoretical framework articulated so far allows us to formulate a number of predictions. If structure-mapping holds water as an account of the empirical basis of iconicity, and if iconicity ratings capture people’s subjective feelings of form-meaning fit, we expect the following to hold:

1. Structural correspondences (Study A) should predict both subjective iconicity ratings (Study B) and objective guessability. Higher cumulative structural correspondences should result in higher subjective iconicity ratings and higher objective guessability.
2. Subjective iconicity ratings (Study B) should correlate with the objective guessability of ideophones. Words rated higher in iconicity should be words that are easier to guess.
3. Ratings and structural correspondences will vary across semantic domains in a way that depends on the affordances for iconic mappings. Specifically, we predict Sound and Motion ideophones (in that order) to show more cumulative structural correspondences (Study A) and to receive higher average iconicity ratings than other domains (Study B).

That the 239 ideophones come from two independent prior studies with different experimental designs and participants provides us with a form of internal replication: if our predictions hold across both sets, this is some reason to trust their generalizability and robustness. However, we also foresee limitations. Our coding of structural correspondences is necessarily selective and unlikely to capture all available cues. For this reason, our predictions are mostly directional: we expect especially strong convergence on the higher end of scores.

More fine-grained patterns and potential mismatches leave room for a set of exploratory analyses that we report in a separate section. We expect that the residue of apparently iconic words missed by our iconicity coding will offer a mix of less widely attested associations and experimental confounds and other noise. The mismatches between measures can be exploited to improve our grasp of iconicity and to point to areas that are not yet well understood. So our approach of methodological triangulation contributes theoretical grounding as well as a tool for empirical discovery.

## METHODS AND MATERIALS

The materials in this study consist of 239 audio recordings of ideophones from five non-Indo-European languages that each have a sizable class of ideophones (also called expressives or mimetics): Japanese (Japonic), Ewe (Kwa, Niger-Congo), Korean (Koreanic), Semai (Aslian, Austroasiatic) and Siwu (Na-Togo, Niger-Congo). The total set combines ideophones tested in two previous studies: 38 Japanese ideophones from a guessing task reported in Collabra (Lockwood et al., 2016), and 201 ideophones from all five languages, from a task reported in Language (Dingemanse et al., 2016). The ideophones in the Language study were selected from five broad semantic domains represented in many ideophone inventories: Sound, Motion, Shape, Colour and visual appearance, and Texture. The ideophones in the Collabra study were not so selected, but 21 of them can be assigned to one of these domains, leaving 17 uncategorized.^3^

The Collabra experiment was a two alternative forced choice task done by 40 Dutch participants, which resulted in a set of ideophones found to be guessed well above chance level at 73% accuracy on average (range 40%–97%). The Language experiment was a two alternative forced choice task done by 80 Dutch participants, which resulted in a set of ideophones found to be guessed moderately above chance on average at 57% accuracy (range 10%–95%), with performance significantly differing by semantic domain and by audio version. The baseline difference in performance between these studies can be attributed to different methods of choosing foils (opposites for Collabra, randomly sampled alternatives for Language) and possibly also audio quality of stimuli (lab recordings for Collabra; field recordings for several of the languages in the Language study). Both studies present a range of outcomes that we can use to gain insight into what makes some words more easy to guess than others. In short, we aim to predict *which* ideophones float to the top, and to explain *why*.

**Figure 2.**
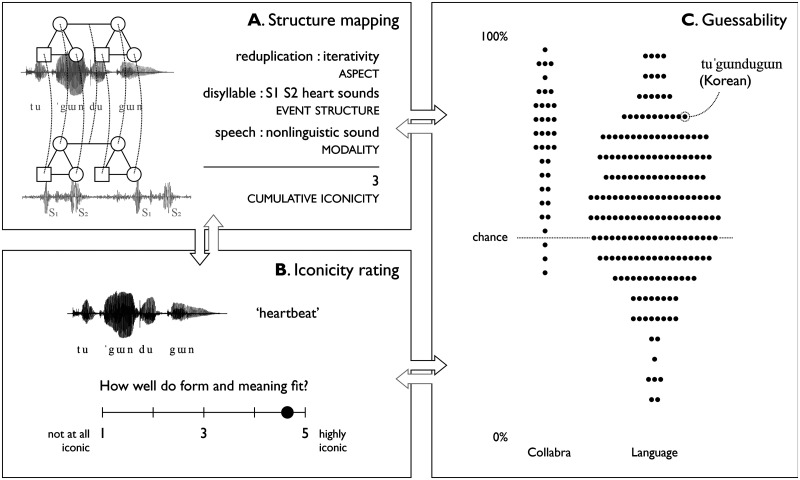
Overview of the triangulation method. Structure mapping grounds the notion of iconicity, ratings capture subjective form-meaning fit, and guessability provides a baseline of guessing accuracy. For instance, Korean *tuˈgɯndugɯn* ‘heartbeat’ features at least three levels of iconic form-meaning correspondences (A). This predicts it should be rated as highly iconic (B) and should be more guessable than words with fewer detected correspondences (C). Consistent with this, it was rated 4.8 for subjective iconicity and guessed at 80% accuracy. Arrows indicate how measures predict (black) and inform (grey) each other.

We do this in two independent ways. In Study A, we systematically weigh up attested iconic correspondences. The more iconic structure mappings attested in a given word, the stronger its overall iconicity, i.e., the resemblance it exhibits between aspects of form and meaning. For example, we can find at least three levels of structural form-meaning correspondences in Korean *tuˈgɯndugɯn* ‘heartbeat’: its overall reduplication can be seen to express iterativity, its lopsided syllabic structure (*tuˈgɯn*) maps onto the first and second heart sounds, and it likely gets further mileage out of using speech to imitate non-linguistic sound (Figure 2A). In Study B, we collect iconicity ratings, giving us a measure of people’s subjective feelings of the degree to which form and meaning resemble each other. Both of these can be related to each other and to the empirical baseline of guessability established in prior work (Figure 2).^4^

Materials for both studies are available in the following OSF repository: https://osf.io/zpcvs/.

## STUDY A: CODING ICONICITY

In this study we systematically surveyed structural correspondences between form and meaning. We inductively designed a coding scheme, tested its consistency and refined it over multiple stages. The point of departure was a set of cross-linguistically attested structural correspondences based on classic work on ideophones (Diffloth, 1972; Jakobson & Waugh, 1979; Westermann, 1927) and more recent surveys (Dingemanse et al., 2020; Johansson et al., 2020; Vigliocco & Kita, 2006). We sharpened the definitions of form and meaning features in light of our cross-linguistic dataset. For instance, while a preliminary version of the scheme included a single form feature of reduplication, we observed both full and partial reduplication in our collection, with cross-linguistic evidence for a difference in corresponding meanings (Akinbo, 2023; Diffloth, 1972), so we distinguished those.

In a second stage, two of the authors coded all ideophones for the form and for the meaning features independently. Average raw percentage agreement was 93% for form features (mean Cohen’s *κ* = 0.8) and 86% for meaning features (mean Cohen’s *κ* = 0.6). However, in both the form and meaning domains there were several features that did not meet the threshold of substantial agreement (Cohen’s *κ* > 0.6). We revisited the least consistently coded categories to recalibrate coders’ understanding of the features, sharpened coding instructions and in some cases renamed coding categories to reflect the refinements.

This led to the third stage: the formulation of the final coding scheme. After this revision we discussed all inconsistent cases to reach a consensus and recoded accordingly. The result is (i) a coding scheme that should be specific and reproducible and (ii) a canonical, ground truth set of calibrated coding data for our set of 239 ideophones. The supplementary materials provide the iterations of the coding scheme, the full details of the coding and consistency procedures, and the first (uncalibrated) and second (calibrated) versions of the coded data.

The final coding scheme consists of 9 form features and 7 meaning features, coded separately and subsequently combined according to predefined structural correspondences (Table 1, and see full coding scheme in online materials). Coding form and meaning separately is a maximally conservative approach that shields against a form of confirmation bias whereby one’s subjective feeling of iconicity may influence the search for structural correspondences. Some of the form and meaning feature pairs are mutually exclusive (for instance, a form cannot be both reduplicated and monosyllabic, and a meaning cannot be both repeated and taking place only once), so we end up with 5 broad types of structural correspondences as shown in Table 1.

**Table 1.** Five broad structural correspondences and their operationalization in the coding scheme, with examples to illustrate congruent form-meaning associations.

**Congruency**	**Form features**	**Meaning features**	**Example**
Aspect	F_redup	M_distribution	Ewe *tyatya* ‘walking fast’
	F_monosyllabic	M_punctual	Ewe *kpa* ‘sound of a slap’

Magnitude	F_weight_voice	M_weight	Siwu *fiϵfiϵ* ‘silky smooth’, Ewe *dzɔvuu* ‘bloated’
	F_weight_vowel		Japanese *don* ‘thud’, Ewe *kpiii* ‘light greyish’
	F_weight_tone		Semai *reŋ:* ‘thin glass breaking’

Modality	spoken word	M_sound	Korean *kkilkkil* ‘giggle’

Length	F_finallength	M_long	Siwu *sɔdzɔlɔɔ:* ‘ellipsoid, elongated shape’
	F_closedsyllable	M_abrupt	Japanese *pon* ‘plop’

Irregular	F_redupmod	M_irregular	Korean *churongjurong* ‘hanging in clusters’

The resulting five broad structural correspondences can be summarised and linked to prior cross-linguistic observations as follows. *Aspect* covers cases of Gestalt iconicity, where the form of a word maps onto the form of the depicted event, for instance reduplication for iteration or monosyllabicity for an event that occurs only once (Kouwenberg & LaCharité, 2001). *Magnitude* covers cases of relative iconicity, where related forms map onto related meanings: for instance, a light versus dark vowel contrast in form mapping onto a light versus heavy or thin versus thick contrast in meaning (Westermann, 1927). *Modality* covers cases of imagic iconicity, where form and meaning sharing the same sensory quality; in this case, sound (as in spoken words that evoke non-linguistic sound). *Length* covers cases where extension in form maps onto length in space or time (Nuckolls, 1996). *Irregularity* covers cases where partial reduplication in form maps onto a sense of irregularity in meaning (Diffloth, 1976). There is no claim that these correspondences are exhaustive or that they cover all iconic aspects to be found in ideophones or other words. The aim is to capture a selection of prominent and widely attested structural correspondences in a way that can consistently be used to assess the quality and quantity of iconicity in individual words.

As the 5 broad types of structural correspondences are logically independent, they can be summed to create a cumulative iconicity score per word as shown in Table 2. This provides us with a way to turn observable facts about the forms and meanings of words into principled statements about their relative degree of iconicity which may in turn be connected to other measures of iconicity.

**Table 2.** Examples of ideophones spanning the distribution of highest to lowest cumulative iconicity scores. Congruency measures are ordered by proportion of occurrence, with ASPECT highest (attested in 98 ideophones or 41%) and IRREGULARITY lowest (attested in only 10 ideophones or 4%).

**Example**	**Aspect**	**Magnitude**	**Modality**	**Length**	**Irregular**	**Total**
% attested	41%	26%	21%	9%	4%	
Ewe *gbììm* ‘explosion’	☑	☑	☑	☑	☐	4
Kor *k’ungk’ung* ‘heavy person walking’	☑	☑	☑	☐	☐	3
Jpn *chirachira* ‘flickering’	☑	☑	☐	☐	☐	2
Jpn *buruburu* ‘shivering’	☑	☐	☐	☐	☐	1
Sem *rŋʔɒɒŋ* ‘round hole’	☐	☐	☐	☐	☐	0

### Results

**Figure 3.**
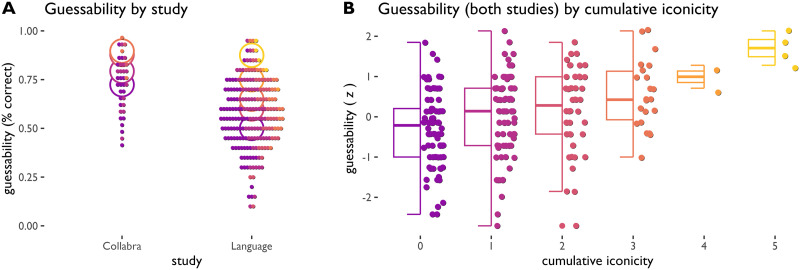
Guessability of ideophones by study and by cumulative iconicity. Every dot represents a single ideophone. A: Guessability in terms of % correct guesses per ideophone, showing the two studies differ in average guessability and in the distribution of variation, but show the same relation between guessability and cumulative iconicity; circles represent mean guessability per cumulative iconicity level, colour represents cumulative iconicity (lighter = higher). B: Guessability (z-scored) across both studies, showing increase in mean guessability and improved diagnostic strength as cumulative iconicity goes up: ideophones with multiple identified iconic mappings are easier to guess.

We find that cumulative iconicity predicts guessability: ideophones with higher cumulative iconicity scores tend to be guessed more accurately (Figure 3). The correlation is found in the Collabra study (Pearson’s *r* = 0.46, *p* < 0.0001) as well as in the Language study (Pearson’s *r* = 0.35, *p* < 0.0001, *p-*values Bonferroni-corrected for multiple comparisons). As predicted, the association is partial: higher cumulative iconicity results in higher guessability and a more narrow range of guessability scores, while ideophones with lower cumulative iconicity represent the full range of variability (indicating our coding scheme only picks up a subset of apparently available iconic cues).

To investigate the relation between cumulative iconicity and guessability in more detail while controlling for differences in study design and a small number of repeated items across studies, we use mixed effects modelling (Bates et al., 2015). We construct a mixed effects model of guessability as a function of cumulative iconicity, with study as a fixed effect, ideophone as a random effect, and cumulative iconicity as a continuous variable (range 0 to 5). A model with cumulative iconicity as a fixed effect achieves a fit that is significantly better than a null model without it (*χ*^2^(1) = 30.3, *p* < 0.0001, log likelihood difference 15.15). The fixed effect of cumulative iconicity is estimated at 0.182 in log odds. Under the simplifying assumption that the distance between iconicity measures is the same at every scale point, this would mean that every step up in cumulative iconicity makes it 6% more likely that an ideophone is guessed above chance.

We can assess the relative weight of different iconic congruency measures using model comparison. We build mixed effects models by adding the congruency measures in rank order of attestation in the coded data (aspect > magnitude > modality > length > irregularity). We find that aspect, modality and length each independently improve model fit. Compared to a null model that represents only the baseline difference between studies and possible item effects, the fuller model including aspect, modality and length fares significantly better (*χ*^2^(3) = 42.59, *p* < 0.0001, log likelihood difference 21.3). Expressed in percentage points boosts to guessing accuracy relative to 50% chance level, the model estimates of the fixed effects corresponding to each of the measures are as follows: aspect 7%, modality 7%, length 17%. In other words, when reduplicated forms combine with iterative meanings, this makes the ideophones in our sample 7% easier to guess; and when long final vowels combine with durative meanings or closed syllables with events that end abruptly, this makes ideophones in our sample 17% easier to guess.^5^

#### Cumulative Iconicity by Semantic Domain.

Sound and Motion ideophones show more cumulative structural correspondences than the other three domains, as predicted (Welch’s *t*(170.68) = 7.61, *p* < 0.0001, *d* = 1.07, 95% CI [0.79, 1.36]). This holds even when excluding the modality feature, which could be said to unduly privilege sound ideophones (Welch’s *t*(189.7) = 6.48, *p* < 0.0001, *d* = 0.89, 95% CI [0.61, 1.17]). Descriptively, the order of domains by decreasing cumulative iconicity is Sound > Motion > Colour/Visual > Shape > Texture.

## STUDY B: RATING ICONICITY

In this rating study we collected iconicity ratings for 239 ideophones.^6^ In total, 78 native Dutch speakers participated in this study. They were recruited mostly via online platforms. Two participants turned out to have knowledge of one or more of the languages in the survey (Japanese and Korean). These two were excluded from the analysis, keeping things methodologically consistent with the Collabra and Language guessability studies, in which participants had no knowledge of the languages in question.

For the remaining 76 participants we calculated person-total correlations (Curran, 2016; Donlon & Fischer, 1968) using a method made available by Motamedi et al. (2019). Person-total correlations can identify careless responses in online data collection by calculating “how consistent any given person is, relative to the expected patterns generated by all other persons” (Curran, 2016: 12). One participant showed a negative person-total correlation, which according to Curran (2016) may indicate a careless responder. Inspection of this participant’s data showed they had indeed given the same answer to every question. We removed this participant from the dataset, resulting in a total of 75 participants to be included in the analysis. These 75 participants (64% female, 35% male, 1% other) are between 19 and 77 years old (*M* = 37;9, *SD* = 17;6).

The iconicity ratings were collected via the online survey platform Qualtrics^7^. Before the experiment started, the term ‘iconicity’ was explained to the participant in lay terms. The instructions were partly based on prior iconicity rating studies (Kwon, 2018; Perry et al., 2015; Winter et al., 2017). We aimed to further optimize these instructions by making them more concise, and by defining iconicity as ‘when a word and its meaning resemble one another’ rather than ‘when a word sounds like what it means’ in order to stress the bidirectional nature of iconicity and to avoid privileging the auditory dimension. The full instructions are included in the supplementary materials.

The ideophones were quasi-randomly divided over four lists of 60, with the number of ideophones per semantic domain and language kept as equal as possible. An overview of the number of items per category and language for all four lists is provided in the supplementary materials. After a practice item, one of the four lists was presented to each participant. This resulted in a total of 18 to 20 raters for each ideophone, which is nearly twice the advised minimum of 10 raters per ideophone (Motamedi et al., 2019). Items were presented in randomized order so that possible fatigue effects were minimized.

For each item, participants listened to the audio recording of an ideophone (which could be played multiple times) and read its Dutch translation. They were then asked to rate the iconicity of this ideophone on a scale of one to five, where one stood for ‘not iconic at all’ and five for ‘highly iconic’. In contrast to Perry et al. (2015), we decided not to include a scale below zero for ‘anti-iconicity’, because in their study, the negative part of the scale was used less frequently as well as less consistently (Motamedi et al., 2019: 10). The scores were given on a slider and rounded to one decimal. The experiment took around 10 to 15 minutes to complete.

### Results

We find that the mean iconicity rating across all ideophones is 2.93, with a standard deviation of 1.30).^8^ Some ideophones were consistently rated as highly iconic, like Siwu *kpa* ‘tock, sound of dry impact’ which was rated between 4 and 5 by every rater (*M* = 4.65, *SD* = .38). Other ideophones were very consistent in getting low ratings, like the Semai *plɒ̃s* ‘sound of someone’s breathing when sleeping’, which was never rated higher than 2 (*M* = 1.33, *SD* = .43), or the Japanese *boo boo* ‘fire burning’, always scoring between 1 and 2.4 (*M* = 1.39, *SD* = .47). These extremes represent quite a small part of the dataset, as only five ideophones were stuck in the lower half of the scale (i.e., never rated 3 or above), and six in the upper half (only rated higher than 3). Most ideophones varied quite a lot in their rating scores, such as the Japanese *zuratto* ‘state of things being arranged in a line’, ratings of which ranged from 1 to 5 (*M* = 2.41, *SD* = 1.50).

**Figure 4.**
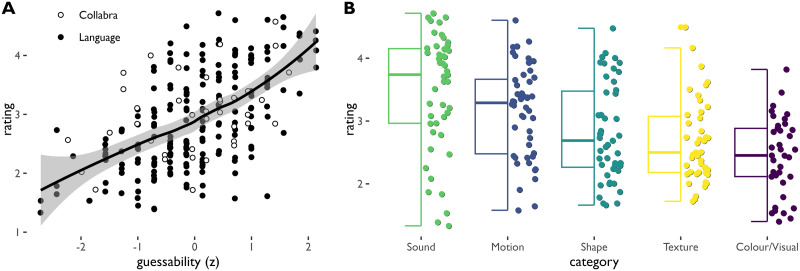
Iconicity ratings by study and by semantic domain. Each dot represents an ideophone. A. Relation between mean iconicity rating and guessability (normalised z-score). Each dot represents a single ideophone. Smoothed conditional mean shown by a loess function with 0.95 credibility interval in the shaded area. B. Distribution of iconicity ratings in relation to semantic domains.

There is a fairly strong positive correlation between iconicity ratings and guessability scores, as illustrated in Figure 4A (Pearson’s *r* = .57, *t*(237) = 10.56, *p* < .0001). This is in line with the prediction that words rated higher in iconicity also tend to be guessed more accurately. When we zoom in where the two methods deviate, it appears that some ideophones with very low iconicity ratings were in fact highly guessable. Conversely, there were no highly rated ideophones with a guessability lower than 45%. There were only four ideophones for which the difference between the z-scores was larger than 2; these belong to the semantic domains Colour/Visual and Texture. For the two Colour/Visual ideophones, both from Semai, the ratings were much lower than the guessing scores, while for the two Texture ideophones, from Semai and Korean, this was the other way around.

#### Iconicity Rating by Semantic Domain.

Figure 4B shows iconicity ratings as a function of semantic domain. As expected, ideophones from the categories Sound (*M* = 3.47, *SD* = 1.33) and Motion (*M* = 3.18, *SD* = 1.24) were rated the highest on average. Especially for Sound, the ideophones show a high density in the upper part of the scale; however, the spread of the ratings is quite large, meaning there are also sound ideophones with much lower ratings. For the categories Shape (*M* = 2.87, *SD* = 1.28), Texture (*M* = 2.68, *SD* = 1.21), and Colour/Visual (*M* = 2.45, *SD* = 1.21), iconicity ratings are mostly below 3 and concentrated in the lower part of the scale.

The order of semantic domains by decreasing iconicity rating is Sound < Motion < Shape < Texture < Colour/Visual. This order corresponds to an implicational hierarchy proposed in McLean (2020): Sound < Movement < Shape < Texture < Other sensory perceptions. This suggests that the most ‘basic’ semantic categories of ideophones (according to the hierarchy) are also judged to be the most iconic ones.

#### Predicting Rating by Cumulative Iconicity.

**Figure 5.**
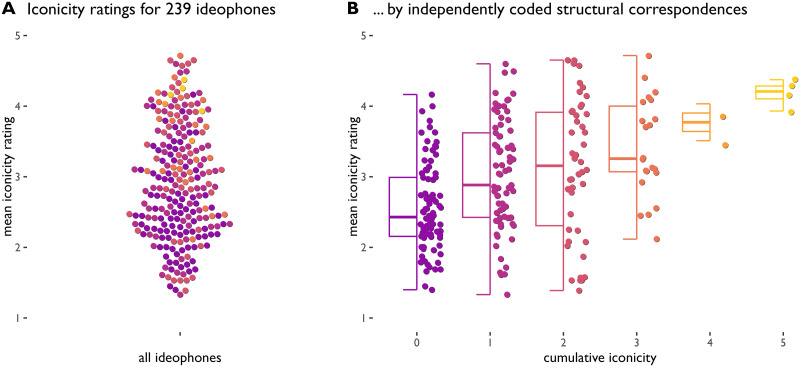
Iconicity ratings (every dot represents a single ideophone). A: Distribution of mean iconicity ratings. B: Distribution of ratings by cumulative iconicity, showing a stepwise increase in iconicity ratings and a narrowing distribution of variance as cumulative iconicity goes up.

A correlation test suggests that cumulative iconicity predicts subjective iconicity ratings (Figure 5): ideophones with higher cumulative iconicity scores tend to be rated higher in iconicity (Pearson’s *r* = 0.40, *p* < 0.0001, and see Figure 5B). As predicted, the association is strongest in the higher regions and not bidirectional: we can predict iconicity rating from cumulative iconicity, but not the other way around.

We construct a mixed effect model of iconicity ratings as a function of cumulative iconicity, with study as a fixed effect and ideophone as a random effect. A model with cumulative iconicity as an additional fixed effect achieves a fit that is significantly better than a null model without it (*χ*^2^(1) = 44.1, *p* < 0.0001, log likelihood difference 22.04). The fixed effect of cumulative iconicity is estimated at 0.30. Under the simplifying assumption that the distance is the same at every scale point, this would mean that every step up in cumulative iconicity makes for a .30 boost in iconicity rating counting from the intercept of 2.76 (recall that the scale runs from 1 = non-iconic to 5 = highly iconic).

We can assess the relative weight of the iconic congruency measures using model comparison as above. We find that aspect, modality and length each independently improve model fit. Compared to a null model that represents only the baseline difference between studies and possible item effects, the fuller model including aspect, modality and length fares significantly better (*χ*^2^(3) = 69.61, *p* < 0.0001, log likelihood difference 34.8). Expressed in terms of points on the 1–5 rating scale, the model estimates corresponding to each the congruency measures are as follows: length +0.84, aspect +0.48, modality +0.26. This is the same relative weighting of each measure as in the model predicting guessability from the iconicity congruency measures.

## EXPLORATORY ANALYSES

**Figure 6.**
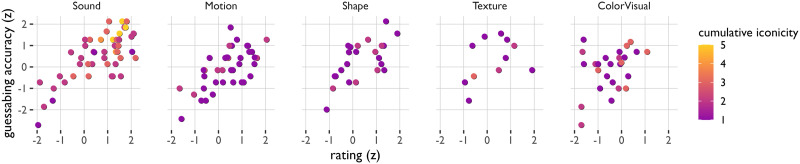
Relation between guesses, ratings, and cumulative iconicity by semantic domain (every dot represents a single ideophone).

One of the key benefits of the methodological triangulation is that it offers an opportunity to identify relative strengths and weaknesses of different measures. In this section, we explore how divergences between the three measures shed light on their limitations and identify opportunities for future research. Figure 6 provides a general overview of the agreement between the three measures, for each semantic domain separately. The online supplementary materials also offer an interactive visualization that makes it possible to explore these measures at the level of single ideophones.

First, we find strong correlations between guessing scores and iconicity ratings for the domains Sound (Pearson’s *r* = 0.74, *p* < 0.0001), Motion (Pearson’s *r* = 0.66, *p* < 0.0001), and Shape (Pearson’s *r* = 0.64, *p* < 0.0001), but not Texture (Pearson’s *r* = 0.38, *p* = 0.24) or Colour/Visual ideophones (Pearson’s *r* = 0.34, *p* = 0.09). Again, this follows the ordering of the implicational hierarchy, with stronger correlations found in more ‘basic’ semantic categories for ideophones. In the case of the Texture and Colour/Visual ideophones, it seems the weaker correlations were primarily driven by ideophones that were guessed fairly well yet rated low in iconicity. This suggests that the Dutch participants underestimated the iconicity of these ideophones when performing the rating task, even though they were able to guess them fairly well in the guessing experiments. This is probably because these are not typical domains for iconic words in Dutch, even though they are common domains for ideophones cross-linguistically. A similar result was reported in another study in which English participants rated the iconicity of Japanese ideophones (McLean et al., 2023).

We also evaluated the coding scheme on a domain-level basis, to see which domains our qualitative measure of iconicity targeted best. Table 3 shows the mean cumulative iconicity score by semantic domain and feature coded, using the coding scheme described in Study A: Coding Iconicity. Higher values are shown in orange, and lower values in blue. A mean score of 1 indicates that every ideophone from that semantic domain was coded for that feature, while a mean of zero indicates that no ideophones from that semantic domain were coded for that feature.

**Table 3.** Mean cumulative iconicity score by semantic domain and feature coded, using the coding scheme described in Study A: Coding Iconicity.

**Feature coded**	**Semantic domain of ideophone**					
	*Sound*	*Motion*	*Shape*	*Texture*	*Colour/Visual*	*Other*
modality	1	0.04	0	0	0	0
aspect_iterative	0.53	0.73	0.04	0.09	0.43	0.18
irregular	0.02	0.06	0.04	0.02	0.08	0
length_closure	0.28	0.02	0	0	0	0
length_punctual	0.28	0	0	0	0	0
length_long	0.02	0.04	0.08	0	0	0
magnitude_voice	0.17	0.06	0.23	0.14	0.32	0.12
magnitude_vowel	0.17	0.06	0.23	0.070	0.27	0.06
magnitude_tone	0.06	0.02	0	0.023	0.08	0

Generally, we can see that sound ideophones were coded for the most features, including the features modality (which only applies to sound ideophones; see Table 1), length_closure (e.g., Japanese *pon* ‘plop’), and length_punctual (e.g., Ewe *kpa* ‘sound of a slap’). However, motion ideophones were more often coded for the feature aspect_iterative (e.g., Ewe *tyatya* ‘walking fast’). After sound, the coding scheme was best at capturing the iconicity of ideophones from visual domains like Shape and Colour/Visual. In particular the features magnitude_voice and magnitude_vowel were most relevant to visual ideophones (e.g., Ewe *dzɔvuu* ‘bloated’, *kpííí* ‘light greyish’). In comparison, the coding of iconic features for ideophones in the domain of Texture was very poor. Although several texture ideophones were guessed very well (e.g., Korean *mullŏngmullŏng* ‘yielding, elastic’, and Japanese *fuwafuwa* ‘fluffy’), the current coding scheme could not tell us why. This highlights the need for more research into the mechanisms of iconicity in domains beyond sound and vision, with texture in particular standing out as a domain to focus on.

As noted above, the coding scheme is geared towards cross-linguistic attestation and reproducibility, so it picks up only a subset of possibly available iconic cues. This is clear from Figure 3B and Figure 6B, where lower cumulative iconicity scores go together with a wide distribution of guessability *z*-scores and ratings. Interpreting mismatches between our bottom-up measure of iconicity and the guessability and ratings data would require careful qualitative follow-up work. We expect at least the following factors to play important roles in explaining mismatches: 1) iconic cues that fall outside our coding scheme, 2) aspects of experimental design, including confounds of foils (in guessing tasks) and written forms (in rating tasks); and 3) confounds of linguistic knowledge in guessing and rating tasks.

Some of the congruency measures have relatively small diagnostic power in our dataset. In most cases this is because form-meaning pairs are simply too infrequent to tell whether they work or not. For instance, the form-meaning pair intonation+weight (which contributes to the magnitude measure) applied to only 8 out of 239 items. While all of these are consistent with Westermann’s original observation that high tones map onto small and light meanings (e.g., Ewe *lúmɔlúmɔ* ‘the scuttling of small animals’, Siwu *gélégélé* ‘shiny’), the numbers are too small to make a statistical difference. Similarly, irregularity (operationalized as a mapping between modified reduplication and a sense of unevenness) was found in only 10 out of 239 items (e.g., Korean *ult′ungbult′ung* ‘bumpy surface’), so more data would be needed to gauge its diagnostic power. It is also possible that this mapping would benefit from including other forms of irregularity like alternating vowels or tones.

## DISCUSSION

We have built bridges between three kinds of approaches to iconicity: systematic qualitative work on observed structural correspondences; classic experimental designs targeting the guessability of word meanings based on word form; and collections of iconicity ratings. Each of these three offers its own angle on iconicity, and by combining insights and data from all three we can learn more about the operations of iconicity in natural language.

We used an inductively based coding scheme to identify structural correspondences in 239 ideophonic words from five languages. Coding was done for form and meaning independently, and the results provide insight into the quality and quantity of iconic mappings that occur in ideophones across languages and across semantic domains. This made it possible to quantify *degree* of iconicity and relate it to outcomes of guessing and rating tasks. We found that coding-based cumulative iconicity is strongly predictive of both guessability and of iconicity ratings: ideophones with higher cumulative iconicity scores were easier to guess and received higher iconicity ratings. As predicted, lower cumulative iconicity scores did not always go with lower guessing and rating scores; this reflects the relatively coarse-grained and non-exhaustive nature of the coding scheme.

Our approach helps open up the black box of iconicity in several ways. At its most general, our approach offers a way to identify the structural form-meaning correspondences that likely underlie people’s guesses of word meaning and ratings of iconicity. We have documented our coding method in some detail to show that it is possible to provide theory-based explanations and predictions for experimental and psycholinguistic measures of iconicity. More specifically, our approach reveals a number of concrete structural correspondences that recur across unrelated spoken languages and across diverse semantic domains. The robust predictive power of most of the structural correspondences we identify indicates that they can be applied to other spoken languages and that they can be used to generate predictions about degrees of iconicity for any set of words.

A key feature of our work is its basis in data from natural languages, rather than made-up words. Even if nonce words like *mil/mal*, *maluma/takete*, and *bouba/kiki* have played important roles in creating awareness of the possibility of iconic form-meaning associations in (spoken) language, they vastly underrepresent the breadth and diversity of forms and meanings attested in the world’s languages. Here we have tried to bring in some of this diversity by considering data from 5 spoken languages from around the world, across at least 5 semantic domains.

### Limitations

Our approach in this study has been limited to spoken languages, and some of the form elements that make up the structural correspondences are modality-specific (e.g., by referring to vowels, voicing, or syllable structure). However, the method is fully generalizable to other modalities; indeed this is why we have chosen to describe the correspondences themselves in modality-agnostic terms like modality, magnitude, aspect, length, and irregularity.

Another limitation of our approach is its focus on cross-linguistically recurrent mappings. There is in iconicity studies always a tension between the search for general patterns of form-meaning association and language-specific, community-specific or even individual iconic associations. This tension is not easily resolved and therefore all the more important to recognize. For instance, here we focus on cross-linguistic attestation because we aim to predict guesses and ratings of ideophone meanings from a bunch of languages by non-speakers of those languages. One consequence is that we chose not to focus on form or meaning features that applied to only some of the languages in our sample, even if we were aware of their relevance—as for instance tone in West-African languages (Akinbo & Bulkaam, 2024; Westermann, 1927) and subtle vowel alternations in Korean (Kim, 1977; Kwon, 2018). If instead one wanted to explain or predict first language acquisition in, say, Japanese, then it would make sense to derive cumulative iconicity from a more detailed and partially language- specific coding scheme (e.g., one based on insights from Hamano, 1998); the prediction would be that this would correlate better with, for instance, ratings by native speakers. In short, how one collects measures of iconicity should be motivated by how one wants to use them; there is no single right way.

Not all correspondences had equal predictive power in our dataset. The most robust across both guessing and rating tasks were aspect, modality and length. Exploratory analyses brought to light considerable diversity across semantic domains, and also revealed some blind spots of the coding scheme. For instance, iterativity was more common in the motion and colour domains, whereas punctuality was more often a feature in the sound domain. Also, several texture ideophones were guessed very well and also rated highly, but the coding scheme provides no direct insight into why. Probing the texture domain on its own in a more detailed investigation would likely pay off; recent work provides insights into relevant form and meaning features (Akita et al., 2024).

### Future Work

The data and methods supplied here offer a number of directions for future work. First, the empirical results have utility beyond the predictive and explanatory uses to which we have put them here. Fine-grained analyses of specific semantic domains will likely bring to light further form-meaning associations. Likewise, deep dives into the ideophone systems of specific languages or groups of languages will likely reveal mappings that may not be universally attested but that are nonetheless recognizable and recurrent: a consequence of the pluripotentiality of iconicity (Winter et al., 2019).

The triangulation method can also be used more systematically in the other direction, using guessing and rating studies to identify possible gaps and missed structural correspondences. For instance, a systematic study of all ideophones that score highly in guessing and/or rating tasks should bring to light aspects of form and meaning that underpin people’s associations. Vice versa, studies of structural complexity in iconic vocabulary (Cuskley, 2013; Klamer, 2002; Mattes, 2018) and of cross-linguistically recurrent associations (Dellert et al., 2021; Joo, 2019) can point to other form-meaning mappings worth investigating.

Moving beyond spoken languages, there is a plethora of work on iconicity in the visual modality that could inspire follow-up work (Cuxac & Sallandre, 2007; Mandel, 1976; Padden et al., 2013). The recent growth in lexical databases for signed languages makes it possible to do cross-linguistic work on signed language lexicons (e.g., Börstell et al., 2016). The methods described here open the way to explore modality-agnostic forms of iconicity in ways that are sensitive to the attested semiotic diversity of languages (Hodge & Ferrara, 2022). We would predict that the three iconicity measures (guesses, ratings, cumulative iconicity) would correlate with one another, but that the correlations would be modulated by modality such that they are strongest in semantic domains that offer most affordances for visual iconicity.

Finally, framing iconicity in terms of analogy-making opens up novel connections to work on structure-mapping and analogical reasoning. Given what we now know about the cumulative structural analogies that underlie intuitions about form-meaning fit, we can ask how the construal and interpretation of iconic mappings is affected by structural affordances of forms and meanings (Clement & Gentner, 1991), how iconic mappings can give rise to processes of abstraction and inference projection (Gentner & Christie, 2010; Verhoef et al., 2015), and how studies of iconicity can benefit from computational models of analogical inference (Blokpoel et al., 2018; French, 2002).

## CONCLUSIONS

There are few principled limits to the ways in which one thing can be “seen as” something else (Hofstadter & Sander, 2013). Nonetheless, there are regularities to the kinds of form-meaning mappings that recur across languages. Here we have used structure-mapping theory (Gentner, 1983) to ground an account of natural language iconicity in lexical items. The focus of structure-mapping is on structure-preserving correspondences between source and target, or, as here, forms and meanings.

Iconicity has long been studied in disparate scholarly fields and with disparate methods: it has been hard to connect insights from fine-grained semiotic analysis to massive rating studies or to laboratory studies of the guessability of iconic words. And yet the promise of unification looms, for across all these fields, iconicity ultimately appeals to our shared intuitions about perceptual analogies between aspects of form and meaning. In this paper we have taken some steps towards bringing these approaches together. We have combined insights from linguistic and semiotic analyses of iconicity with results from guessing and rating tasks to turn shared intuitions into empirically robust statements (McLean & Motamedi, 2024). We have identified a number of structural correspondences in iconic words that are rooted in concrete aspects of form and meaning and whose cumulative occurrence relates in predictable ways to people’s performance in guessing and rating tasks.

Three decades ago Linda Waugh called out the “debilitating premise” of the arbitrariness of the sign, arguing that iconicity is an important part of language (Waugh, 1994). If recent waves of observational and experimental work have solidified the status of iconicity as a linguistic phenomenon (Hodge & Ferrara, 2022; Murgiano et al., 2021; Perniss & Vigliocco, 2014), our goal in this paper has been to move towards a consolidation of insights by demystifying iconicity and by better integrating it into the sciences of language and mind. Our work demystifies iconicity by showing how it is based on well-understood principles of cumulative perceptual analogy-making. It helps integrate iconicity by anchoring it to a tried and tested framework of analogical structure-mapping. The result is an theory of lexical iconicity that is robust and explanatory, that is flexible enough to account for individual and cross-linguistic differences, and that connects with core insights from the cognitive sciences.

Given the affordances of our articulators, the multidimensionality of meaning, and the fractal complexity of the perceived world, iconic signs in natural languages rarely are full-bodied replicas of the sensory scenes they seek to evoke. The good news is that they don’t need to be; they merely need to provide sufficient footholds for structural correspondences between aspects of form and meaning. Here we have shown how such correspondences can explain and predict our intuitions about word meanings, and in the process shed light on the anatomy of iconicity.

## ACKNOWLEDGMENTS

This work was funded by the Dutch Research Council (NWO) through Veni grant 016.154.087 and Vidi grant 016.vidi.185.205 to MD. A first iteration was presented in 2017 and benefited from feedback from audiences at the Iconicity Focus Group workshop (Nijmegen) and the International Congress of Linguists (Cape Town).

## AUTHOR CONTRIBUTIONS

MD and SP carried out the coding study; SP carried out the rating study as part of a BA thesis and authored the corresponding section; BM contributed exploratory analyses and authored the corresponding section; MD wrote the first full draft. All authors contributed to and approved of the final MS.

## Notes

^1^ The notion of non-alignable differences allows us to make a corrective to a prior attempt to operationalize iconicity that also invokes structure-mapping. In an analysis of 248 Chaoyang ideophone codas, Thompson and Do (2019) take the strong position that (barring phonotactically motivated patterns) “every phoneme is meaning-bearing”. This seems hard to defend given the expectations of structure-mapping theory, and limits the predictive power of the account. If every aspect of a word is meaning-bearing, why are some iconic words so much easier to guess than others?^2^ We do not exclude the possibility of less widespread or even language-specific form-meaning correspondences (Diffloth, 1994); but since our aim is to have an account that can make quantitative predictions about degree of iconicity, we focus here on correspondences that are cross-linguistically attested.^3^ To maintain comparability, we opted to use the categories as defined in the larger collection (instead of adding new ones). For the same reason, we have opted to keep the classification discrete, even if ideophones can be seen to fall into multiple sensory domains (Nuckolls, 2019).^4^ Triangulation as a method can be used in multiple directions. Here, we focus on using structure mapping to predict and explain iconicity ratings and guessability. Arrows can also be followed in the reverse direction, for instance by using high guessability or high iconicity ratings as a cue to search for structural analogies. Although we don’t do this here, Figure 2 records this possibility with grey arrows.^5^ These values estimate the separate contributions of one measure while controlling for the others, but they are *not* additive, i.e., they cannot be directly summed to arrive at the effect of cumulative iconicity.^6^ We used one excluded item as a practice item, and another as a filler to get a divisible amount of 240 ideophones. We do not include either of those ideophones in the analyses below to maximise comparability across studies, so the number of reported ratings is 239 ideophones.^7^ https://www.qualtrics.com.^8^ This includes the uncategorized ideophones from the Collabra set. However, this group of uncategorized ideophones does not reflect a clear semantic domain and is much smaller than the other categories (17 ideophones versus around 40 per domain). To make the results more interpretable, we decided to exclude the set of uncategorized ideophones from the rest of the analyses reported here.

## Supplemental Materials

https://doi.org/10.17605/osf.io/zpcvs
